# The Molecular Information About Deadwood Bacteriomes Partly Depends on the Targeted Environmental DNA

**DOI:** 10.3389/fmicb.2021.640386

**Published:** 2021-04-27

**Authors:** Maraike Probst, Judith Ascher-Jenull, Heribert Insam, María Gómez-Brandón

**Affiliations:** ^1^Department of Microbiology, University of Innsbruck, Innsbruck, Austria; ^2^Grupo de Ecoloxía Animal (GEA), Universidade de Vigo, Vigo, Spain

**Keywords:** intracellular DNA, extracellular DNA, environmental DNA, *Picea abies*, deadwood, microbiome, microbial communities

## Abstract

Microbiome studies mostly rely on total DNA extracts obtained directly from environmental samples. The total DNA consists of both intra- and extracellular DNA, which differ in terms of their ecological interpretation. In the present study, we have investigated for the first time the differences among the three DNA types using microbiome sequencing of *Picea abies* deadwood logs (Hunter decay classes I, III, and V). While the bacterial compositions of all DNA types were comparable in terms of more abundant organisms and mainly depended on the decay class, we found substantial differences between DNA types with regard to less abundant amplicon sequence variants (ASVs). The analysis of the sequentially extracted intra- and extracellular DNA fraction, respectively, increased the ecological depth of analysis compared to the directly extracted total DNA pool. Both DNA fractions were comparable in proportions and the extracellular DNA appeared to persist in the *P. abies* deadwood logs, thereby causing its masking effect. Indeed, the extracellular DNA masked the compositional dynamics of intact cells in the total DNA pool. Our results provide evidence that the choice of DNA type for analysis might benefit a study’s answer to its respective ecological question. In the deadwood environment researched here, the differential analysis of the DNA types underlined the relevance of *Burkholderiales*, *Rhizobiales* and other taxa for *P. abies* deadwood decomposition and revealed that the role of *Acidobacteriota* under this scenario might be underestimated, especially compared to *Actinobacteriota*.

## Introduction

Before the advent of sequencing technologies, molecular microbial ecology was severely limited by low-resolution observation of microbial diversity. Nowadays, high-throughput microbiome sequencing provides insightful data on microbial occurrences and dynamics. Nevertheless, limitations still persist. The most common procedures in microbiome studies comprise direct DNA extraction from environmental samples, amplification and subsequent sequencing of marker genes along with the analysis of the resulting compositional dataset. Therefore, next to an adequate experimental design based on a robust sampling strategy, the DNA extracts analyzed largely determine the study’s output. Several types of environmental DNA extracts have been differentiated in the literature as reviewed by Nagler et al. (2018a). Here, we will focus on the three most common DNA types: the total DNA pool and its extracellular and intracellular DNA fractions (Nannipieri et al., 2020). Usually, only the total DNA pool is used. It is extracted directly from the environmental samples using standard protocols based on enzymatic, physical and/or chemical cell lysis and contains both intra- and extracellular DNA. The intracellular DNA fraction is obtained from intact cells contained in the environmental sample; while the extracellular DNA fraction is composed of DNA – no longer present inside cell membranes – occurring in the extracellular environment (Pietramellara et al., 2009; Nagler et al., 2018b). In fact, extracellular environmental DNA can be defined as former intracellular DNA that has been released either actively (active DNA extrusion) or passively (after cell death and consequent cell lysis) into the environment. Methods to sequentially extract the extra- and intracellular DNA fractions of different environmental matrices or biological assemblages are available (Ascher et al., 2009; Wagner et al., 2015; Carini et al., 2016; Nagler et al., 2018a). First, the DNA occurring in the extracellular environment is recovered by gentle washings in alkaline buffer; the remaining pellet is then processed by cell-lysis inducing steps so as to extract the intracellular DNA. Thereafter, both DNA fractions are purified to obtain DNA compatible for downstream analyses. Due to the various terms and abbreviations used for these different DNA types in the literature (reviewed by Nagler et al., 2018a) and to avoid any misunderstanding and misinterpretation of the results, we will not use any acronyms throughout the text.

While the intracellular DNA represents intact and thus, potentially living cells (without the possibility to discriminate between active and dormant cells), the extracellular DNA originates either from cell lysis and/or active secretion (Levy-Booth et al., 2007; Pietramellara et al., 2009; Nagler et al., 2018b, 2020). Regarding the active release of DNA, it is still unknown if DNA is released on purpose or randomly. There is evidence that its secretion into the environment is a common feature of both Prokaryotes and Eukaryotes and might provide selective advantages: Nagler et al. (2018b) reported that the concentration of microbial extracellular DNA is proportional to microbial activity. Moreover, extracellular DNA was found to be a quorum sensing signal and the concentrations of conspecific extracellular DNA were shown to regulate cell growth of bacteria, fungi, algae, plants, protozoa, and insects, e.g., in the innate defense system (Mazzoleni et al., 2015; de Aldecoa et al., 2017; Ferrusquía-Jiménez et al., 2020). However, in addition to its signaling ability, extracellular DNA is relevant as it can foster genetic exchange via natural transformation and serve as a nutrient source (Levy-Booth et al., 2007; Pietramellara et al., 2009; Morrissey et al., 2015; Nagler et al., 2018a).

Extracellular DNA can persist in the environment due to physical protection against enzymatic denaturation and its turnover rate can range from a few hours to several months and years (Nielsen et al., 2007; Nagler et al., 2020), up to extended periods of time (Agnelli et al., 2007). Environmental conditions, such as low temperatures, low pH, and the presence of salt and clay minerals can slow down extracellular DNA degradation and consequently, its persistence and residence time (Khanna and Stotzky, 1992; Pietramellara et al., 2009).

The relevance of extracellular DNA has long been neglected in microbial ecological research, despite it has been found at qualitatively and quantitatively relevant concentrations in a wide range of environments (Dell‘Anno and Danovaro, 2005; Pietramellara et al., 2009; Carini et al., 2016, 2020; Nagler et al., 2018a,b) and despite the different ecological interpretations of the DNA types. By only assessing the directly extracted total DNA pool, consisting of extra- and intracellular DNA, there is the risk of extracellular DNA masking the intracellular DNA within the community analysis. Carini et al. (2016) reported that up to 40% of both prokaryotic and fungal DNA in soil was attributed to extracellular DNA. This led to an inflation of microbial richness measures by up to 55% and a misestimation of relative taxon abundances, including some key taxa for the ecosystem functioning.

Although the different DNA types have been drawing more attention recently, an in-depth screening of the extra- and intracellular DNA fractions compared to the total DNA pool by high-throughput sequencing technologies is still in its infancy. In this study, we offer a particular habitat as an example on how to obtain a comprehensive picture of the microbial community composition. Deadwood is of interest considering that it acts as an important temporary carbon pool in forest ecosystems (Stokland et al., 2012).

The role of bacterial communities in the deadwood habitat is still underexplored compared to fungi (Johnston et al., 2016, 2019; Gómez-Brandón et al., 2020). Yet, the variations among bacterial communities within and between decay stages are poorly studied, as previously emphasized (Hoppe et al., 2015; Kielak et al., 2016b; Lee et al., 2020). Exploring such variations can increase our understanding about the dynamics of the deadwood ecosystem in terms of key players, functional redundant decomposers, and microbial succession. Therefore, we collected natural deadwood logs of Norway spruce (*Picea abies* (L.) Karst.) at different stages of decay – that is decay classes (DCls) I, III and V based on the classification system proposed by Hunter (1990) – in a well described study area in the Italian Alps (Val di Rabbi, Trentino; Petrillo et al., 2015). For an in-depth screening of the deadwood bacteriome, we compared the bacterial community analyzed by Illumina sequencing of the V4 16S rDNA of the directly extracted total DNA pool and its sequentially extracted extracellular and intracellular fractions.

By analyzing these three DNA types we aimed to answer the following questions: (i) Does bacterial richness differ among DNA types and does it increase with progressing decay?; (ii) What can bacteriome information from the intra- and extracellular DNA contribute to the picture drawn by the total DNA pool?; (iii) Is there a masking effect of the extracellular DNA over the intracellular DNA?; and (iv) from our results, can we extrapolate some guidelines that may help in choosing the most suitable DNA type for molecular screening according to the specific research question?

## Materials and Methods

### Sampling

The deadwood samples from Norway spruce (*P. abies* (L.) Karst) were collected in a montane forest at an altitude of 1995 m above sea level (a.s.l.) in Val di Rabbi (Trentino, Italy). A detailed description of the study site is given in Petrillo et al. (2015). The samples were classified *in situ* into DCls I, III, and V based on their visual, geometric and tactile features (Hunter, 1990). Samples from DCl I correspond to hard wood penetrable with a knife for only a few mm. Wood of DCl III is distinctly softer and can be penetrated with a knife to around 1–4 cm. In DCl V, wood is very soft and has a crumbly, instable texture which is easily penetrable with a knife for >10 cm; the shape of the original log is barely recognizable. For each DCl, three samples (biological replicates) were collected from different logs, pre-treated by cut-milling and stored at −20 °C for further analysis according to Gómez-Brandón et al. (2017a).

### DNA Extraction

The total DNA pool was extracted from 0.1 g cut-milled sample using MP Biomedicals DNA Extraction Kit according to the manufacturer’s protocol. To assure a complete disruption of the wood tissue, one 1/400 ceramic sphere (MPI biochemical cat# 6540-424) was added to the lysing matrix. The extra- and intracellular DNA fractions were sequentially extracted by applying the procedure by Gómez-Brandón et al. (2017a). Briefly, 0.1 g cut-milled sample was treated with 500 μl 0.12 M Na_2_HPO_4_ (pH 8) and gently shaken on a horizontal shaker for 30 min; thereafter, the liquid phase containing the recovered extracellular DNA fraction was separated from the solid phase (pellet) containing intact cells by low-speed centrifugation (4 °C, 30 min, 7,500 × *g*). The procedure was repeated twice, for a total of three washing and centrifugation cycles, and the supernatants were pooled. The remaining pellet was processed as described for the total DNA extraction, yielding the intracellular DNA fraction.

All DNA extracts were purified using the GeneClean Kit (components of the MP Biomedical DNA Extraction Kit) and their quality and quantity was checked via agarose gel electrophoresis and fluorimetric measurement (Qubit, LifeTechnologies), respectively.

### Sequencing and Data Analysis

The amplicon library for Illumina Miseq sequencing was prepared in a two-step procedure according to the standard protocol. First, the bacterial 16S rRNA genes of the DNA extracts were amplified using Illumina TruSeq chemistry adapters fused to bacterial 16S primers 515f and 806r (Caporaso et al., 2011). In a second step, samples were barcoded using Illumina TruSeq chemistry and flow cell adapters were attached to the amplicons. The library was sequenced in an Illumina MiSeq PE250 nano run at VBC Vienna. Sequencing data were deposited in SRA under BioProject Number PRJNA 682981.

From demultiplexed fastq files, an amplicon sequence variant (ASV) table was obtained using Dada2 pipeline (version 1.16, Callahan et al., 2016) following the standard protocol provided by the developers on github (version 1.16). Briefly, adapters and primers were trimmed and sequences with ambiguous base pairs were removed from the dataset. Error models were predicted from randomly selected samples from the run and both forward and reverse reads were filtered according to the error profiles. Sequences were merged and filtered for chimeric sequences. Sequences outside a range of 250–254 bp were discarded. A frequency ASV table was generated from the dataset. Taxonomy was assigned to ASVs using SILVA reference database v.138. An ASV-approach was chosen over an approach based on operational taxonomic units (OTUs) for reasons of precision and reproducibility (Callahan et al., 2017).

**FIGURE 1:**
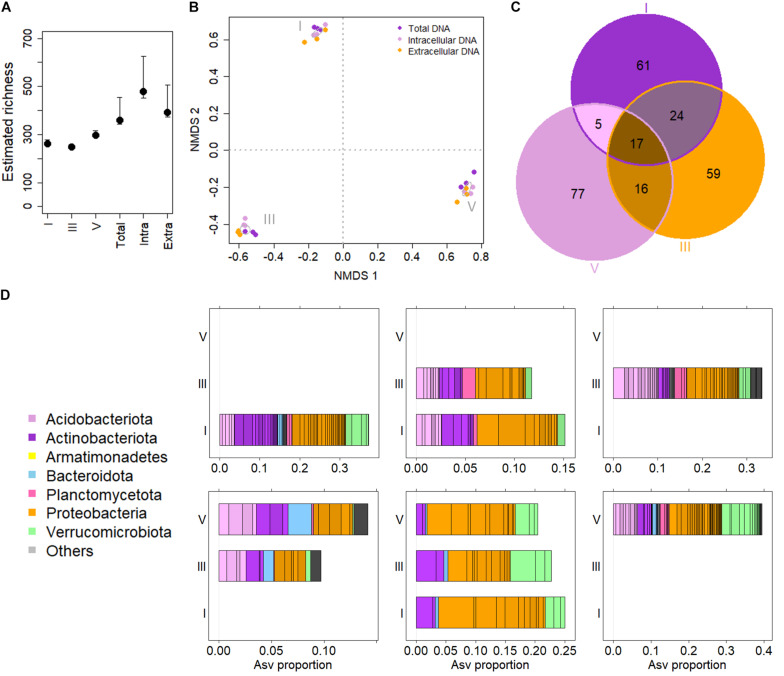
The *Picea abies* deadwood bacteriome across decay classes (DCls). **(A)** Estimated ASV richness comparing DCls (I, III, and V) and DNA types (Extra = extracellular DNA fraction, Intra = intracellular DNA fraction, Total = total DNA pool). **(B)** Non-metric multidimensional scaling of log samples based on Bray Curtis distances. Stress = 0.080. The algorithm converged after 53 iterations. Ellipses were drawn at 95% confidence intervals around group centroids of DCls. **(C)** Venn diagram illustrating ASV compositions of DCls. **(D)** Taxonomic overview of Venn sets **(C)**. The taxonomic composition of ASVs detected only in DCl I is illustrated in the first panel of the top row. The top row second panel shows the composition of ASVs detected in both DCls I and III, but not in DCl V. The top row third panel gives the taxonomic composition of ASVs found only in DCl III. The bottom row illustrates first the taxonomic composition of ASVs detected in both DCls III and V, but not in DCl I. The bottom row middle panel illustrates the taxonomic core microbiome composition of all three DCls. The bottom right panel shows the taxonomic composition of ASVs detected only in DCl V.

The resulting ASV table was filtered prior to further analysis. Only ASVs detected in at least two out of the three samples per sample group were considered, that is the presence of ASVs in the same DNA type extracted from different logs classified into the same DCl. In addition, ASVs with <5 reads per sample group were removed from the dataset. Applying these criteria, the initial number of 1,946 ASVs was reduced to 623 ASVs in the final dataset. Although the removal of ASVs <5 reads reduced the total richness calculated for the samples, it did not affect the comparisons of α-diversity among sample groups. The removal of filtered ASVs also did not affect the compositional distances between samples as inferred from comparing the ordination resulting from both principal component analysis (PCA) of the ilr-transformed compositional data and Non-metric Multidimensional Scaling (NMDS) on Bray-Curtis distances using Procrustes analysis (PCA: *m*^2^_Procrustes_ = 0.0985, correlation of symmetric rotation = 0.950, *p* = 0.001; NMDS: *m*^2^_Procrustes_ = 0.1568, correlation of symmetric rotation = 0.918, *p* = 0.001). Both ordination methods showed comparable results in terms of clustering by sample groups. For illustration purposes (Figure 1B), NMDS was chosen here.

Sequencing depth differed between sampling groups (*p*_Anova_ = 7e-5) (Supplementary Figure 1A). In addition to smaller differences, especially the extracellular DNA fraction of DCl V had a significantly higher number of sequencing reads (prior to filtering and thereafter) compared to all other sample groups (min_extra;_ _DCl__V_ = 14,382, median_extra;_ _DCl__V_ = 14,593, max_extra;_ _DCl__V_ = 22,789 reads after filtering). No differences were found among the other sample groups (min = 4,608; median = 8,007; max = 11,715 reads after filtering). Moreover, there were no significant differences in sequencing depth comparing the groups of main effects, which are DCl (I, III, and V) and DNA type (extracellular, intracellular, and total) (*p*_Anova;_ _DCl_ = 0.124, *p*_Anova;_ _DNA_ _type_ = 0.058).

All data analyses were performed in R 4.0.2 (R Core Team, 2020). Package vegan (Oksanen et al., 2019) was used for ecological multivariate analysis, including NMDS and Procrustes analysis. For PCA, data were ilr-transformed (isometric log ratio) using the R package compositions (van den Boogaart et al., 2020). ASVs with differential abundance among DCls were identified by calculating a generalized linear model using ALDEx2 (Fernandes et al., 2013). Breakaway (Willis et al., 2020) was used to estimate the α-diversity/ASV richness. Data were visualized using lattice (Sarkar, 2008), ggplot (Wickham, 2016), ggtern (Hamilton and Ferry, 2018), and Vennerable (Swinton, 2013). Colors were chosen from RColorBrewer (Neuwirth, 2014).

## Results

### *P. abies* Deadwood Bacteriomes Differ Among Decay Classes (DCls)

#### Bacteriomes Differ Vastly Between DCls and to a Smaller Extent Between DNA Types

Considering the main effects, there were no significant differences in ASV numbers among DNA types and DCls. Among sample groups (DNA types × DCl), ASV numbers differed significantly (*p*_Kruskal_ = 0.007). For both intra- and extracellular DNA fractions the highest numbers of ASVs were detected in DCls I and V, respectively (Supplementary Figure 1B). In contrast, total DNA had the highest number of ASVs in DCl III. However, these differences in the number of ASVs among the sample groups were likely related to the sequencing depth. With this in mind, we further estimated the bacterial ASV richness using breakaway (Willis and Bunge, 2015). The estimated number of ASVs did not vary in terms of estimated richness but was elevated in the intra- and extracellular DNA fractions compared to the total DNA pool (Figure 1A).

The bacterial community composition differed between sample groups (Figure 1B). On a presence-absence distance matrix (Jaccard distance), 48% of the overall variance in the dataset were attributed to differences among the three DCls (*p*_Adonis_ = 0.001). The DNA types were less discriminative (*p*_Adonis_ = 0.001, *R*^2^ = 0.102) in this regard. Interestingly, the interaction effect of DCl and DNA type turned out significant, explaining for 21% of the overall variance (*p*_Adonis_ = 0.001). Using variance partitioning on ilr-transformed abundance data, around 34% of the variance (unadjusted *R*^2^) within the dataset accounted for the DCl (adjusted variance solely attributed to DCl = 24%) and the DNA type (adjusted variance solely attributed to DNA type = 5%) (*p* = 0.001). From an ecological perspective and regardless of Jaccard distance or ilr transformation, the DCls differed in terms of both ASV presence/absence and relative abundance (Figure 1B).

Only a total of seventeen ASVs were detected across the three DCls (core ASVs, Figure 1C). A higher number of ASVs was unique to each DCl (Figure 1C), particularly for DCl V with 77 unique ASVs. This indicates that the bacterial community of this DCl was more dissimilar compared to DCls I and III (15 and 2 unique ASVs, respectively; Figures 1B,C). Both the core and unique ASVs were taxonomically diverse (Figure 1D), and the majority of the core ASVs varied in their differential abundance when comparing the three DCls (11 out of 17 ASVs, Supplementary Table 1).

Given the variation among DCls being larger than regarding DNA types, we analyzed the ecological differences in the compositions of the bacteriomes at the different DCls jointly across all DNA types.

#### Bacterial Community Composition as a Function of *P. abies* Decay Class

**FIGURE 2:**
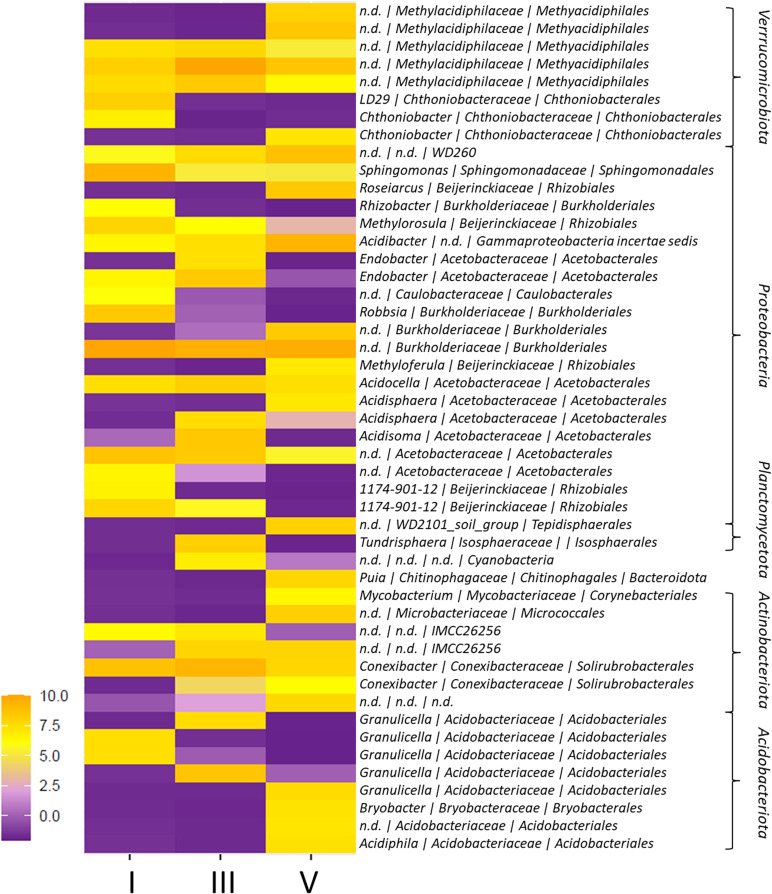
Heatmap of ASVs with differential abundance among decay classes (DCls) I, III, and V. The differential abundance of ASVs among DCls was evaluated using generalized linear modeling (ALDEx2). Only ASVs with a significant, Bonferroni-Holm corrected *p*-value are shown. Taxonomy indicates genus | family | order and phylum annotations.

The majority of sequencing reads was annotated as *Proteobacteria* (>40% of all reads) (Supplementary Figure 2). This phylum also had the highest number of different ASVs (232 out of 623 ASVs), and was primarily comprised by the orders *Acetobacterales* (14% of total reads; 77 ASVs), *Rhizobiales* (8% of total reads; 36 ASVs) and *Burkholderiales* (8% of total reads; 17 ASVs). These taxa also comprised almost all the proteobacterial ASVs unique to each of the sample groups or differentially abundant across DCls (16 out of 20 ASVs; Figure 2; Supplementary Table 1). *Acetobacteraceae* were detected in a relatively high richness across all DCls (>30% of all *Proteobacteria* ASVs), especially in DCl III (12/24 ASVs). The genera *Acidisoma*, *Acidisphaera*, and *Endobacter* revealed to be characteristic for this DCl (Figure 2 and Supplementary Figure 1). *Burkholderiaceae* ASVs (such as *Rhizobacter*) and *Beijerinckiaceae* ASVs (such as *Methylorosula*) were associated with DCl I (Figure 2 and Supplementary Table 1). In DCl V, ASVs with particularly high abundance or ASVs unique to this sample group appeared taxonomically more diverse. Proteobacterial ASVs characteristic for this DCl were annotated as *Roseiarcus* (*Beijerinckiaceae*), *Burkholderia* (*Burkholderiaceae*), and *Acidisphaera* (*Acetobacteraceae*) (Figure 2 and Supplementary Table 1). Interestingly, ASVs with high abundance in DCl V were often lower or not detected in DCl I and vice versa. For instance, the richness of *Rhizobiales* was higher in DCl I (20 ASVs) compared to DCl V (14 ASVs), while a higher number of *Burkholderiales* ASVs was found in DCl V (12 ASVs) with respect to DCl I (9 ASVs).

In addition to *Proteobacteria* (245 ASVs), *Actinobacteriota* (74 ASVs), *Acidobacteriota* (99 ASVs), and *Verrucomicrobiota* (71 ASVs) were detected in all sample groups (>14% of all reads; Figure 1D). In DCl I, the proportion of *Actinobacteriota* reads exceeded the proportions of *Acidobacteriota* and *Verrucomicrobiota* reads. Within *Actinobacteriota*, members of the order *Solirubrobacterales* accounted for the highest number and read percentage (7% of all reads, 29 ASVs). The proportion of *Acidobacteriota* reads increased from DCl I (14% of all reads) to DCl III (19% of all reads), where its proportion was even higher compared to *Actinobacteriota* and *Verrucomicrobiota*. The vast majority of *Acidobacteriota* reads were annotated as *Acidobacteriales* (14% of all reads, 87 ASVs). Especially *Granulicella* was found to be more characteristic for DCls I and III. *Granulicella* ASVs had a significantly higher relative abundance in these two DCls, peaking in DCl III, compared to DCl V (Figure 2 and Supplementary Table 1).

*Verrucomicrobiota* increased in read proportion with increasing DCl (I = 12%, III = 14%, V = 17% of all reads), and exceeded both *Actinobacteriota* and *Acidobacteriota* in DCl V. *Methylacidiphilales* ASVs represented the majority of *Verrucomicrobiota* reads (10% of all reads, 25 ASVs) and were differential in abundance among DCls (Figure 2).

Furthermore, we found a total of 64 *Planctomycetota* ASVs, mainly WD2101 soil group (26 ASVs), and their relative abundance increased with progressing decay (Figure 1D). However, their joint abundance accounted for <5% of all sequencing reads (Supplementary Figure 1).

### Total, Intra- and Extracellular DNA Provide Differential Information About *P. abies* Deadwood Bacteriomes

#### Intra- and Extracellular DNA Fractions Enable a Deeper Level of Ecological Observation in *P. abies* Deadwood

Microbiome analysis by high-throughput sequencing largely depends on which sequences are amplified, implying the DNA type targeted. Consequently, when analyzing the total environmental DNA pool, its proportion of intra- and extracellular DNA are decisive for the results. In our study, the concentrations of intra- and extracellular DNA were comparable within each of the three DCls (Table 1). This was in line with the findings by Gómez-Brandón et al. (2017a), who studied the same deadwood logs and observed increasing yields of these two DNA fractions with progressing decay.

**TABLE 1:** Total, intra- and extracellular DNA concentrations in the three decay classes (DCl I, III, and V).

**Decay class**	**Extracellular DNA [μg g^–1^ dry weight]**	**Intracellular DNA [μg g^–1^ dry weight]**	**Total DNA [μg g^–1^ dry weight]**
I	0.32	0.31	2.75
I	2.13	3.61	5.03
I	0.45	0.45	1.59
III	2.66	3.07	3.89
III	2.59	1.96	4.56
III	1.75	0.90	2.36
V	11.26	7.59	27.86
V	14.11	14.22	36.43
V	15.58	8.65	41.79

**FIGURE 3:**
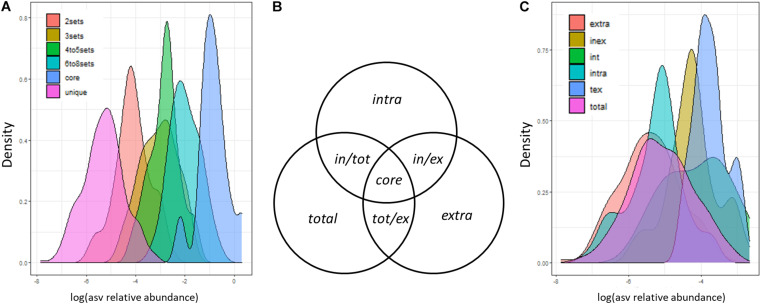
Distribution of ASV sizes (sequence proportions). ASVs were categorized based on **(A)** the number of sample groups (3 decay classes × 3 DNA types) and **(C)** the sets of DNA types (extracellular and intracellular DNA fraction, total DNA pool), in which they were detected. **(B)** Set definitions of DNA fractions, in which ASVs can be detected. These definitions are applied in panel **(C)** and in the text.

Considering the three DCls together with the three DNA types, we expected to detect (i) a small number of ASVs with high relative abundance in all nine sample groups and (ii) a high number of ASVs with lower relative abundance in a single or in a low number of sample groups. Concerning this latter point, we expected the majority of ASVs to be detected in ≤ 3 sample groups, given the big differences among DCls and the dependencies between the bacteriomes of the DNA types derived from the extracts originating from the very same log sample. In line with this, we found that ASVs with higher abundance were detected more often in a higher number of sample groups (Figure 3A). Less than 100 and less than 30 out of 624 ASVs were found in >3 and >6 sample groups, respectively. A total of 285 ASVs (>45%) were unique to one sample group, that is one DNA type of a specific DCl. The majority of these unique ASVs were detected in the extracellular DNA fraction (extracellular: 129 ASVs; intracellular: 64 ASVs; total: 92 ASVs). A total of 126 ASVs (20% of all ASVs) and 114 ASVs (18% of all ASVs) were detected in two and three sample groups, respectively.

From here on, we will refer to ASVs observed solely in the intracellular DNA fraction, extracellular DNA fraction or total DNA pool as *intra*, *extra*, and *total ASVs*, respectively (Figure 3B). ASVs observed in both DNA fractions but not in the total DNA pool will be termed *in/ex ASVs*. Those ASVs observed in one DNA fraction and the total DNA pool will be termed *in/tot* and *tot/ex* ASVs, respectively (Figure 3B). Core ASVs will be those detected across all DNA types (Figure 3B). We found that *in/tot* and *tot/ex* ASVs had a higher relative abundance than *in/ex* ASVs (Figure 3C). In addition, the majority of ASVs which were detected in exactly two different sample groups were also found in the intra- and extracellular DNA fractions of the same DCl (74/126 ASVs). A lower number of those ASVs was found in the total and the extracellular DNA (22/126 ASVs); as well as in the total and the intracellular DNA (15/126 ASVs) of the same DCl. The remaining ASVs detected in exactly two sample groups were found across DCls (15/126 ASVs). Those ASVs detected exactly three times in our dataset were mostly observed in all DNA types of a respective DCl (98/114 ASVs), which underlines the bigger effect of the DCl compared to the DNA type. The remaining 16 ASVs were unique to one DNA fraction, mainly to intracellular DNA and were repeatedly present across the three different DCls.

Given the deeper level of ecological analysis reached in the extracellular vs. intracellular DNA fractions compared to the total DNA pool, we investigated the frequency of those bacterial taxa detected in only one DNA type of the same DCl in comparison to its overall distribution. Therefore, we counted the number of ASVs annotated to a certain bacterial class within each of the nine sample groups and compared the frequencies of class annotations within DCls among DNA types. For a number of bacterial classes, their frequencies differed among DNA types indicating that the taxonomic distribution of ASVs with low relative abundance did not follow the taxonomic pattern of more abundant ASVs (Supplementary Table 2). In DCl I, *Desulfobacterota* ASVs reached higher frequencies in the intracellular DNA fraction compared to the other DNA types. In this DCl, the extracellular DNA did not contain any bacterial classes with higher frequencies in comparison with the total DNA pool. In DCl V, a number of classes showed higher frequencies in the intra- and extracellular DNA fractions than in the total DNA pool. Amongst other phyla, the frequencies of *Acidobacteriota*, *Alphaproteobacteria*, *Bacteroides*, *Desulfobacterota*, *Gammaproteobacteria*, and *Verrucomicrobiota* were increased by at least 1.5-fold in the intracellular DNA and by at least 2-fold in the extracellular DNA fraction compared to the total DNA pool (*p*_Kruskal_ <0.05).

#### Ecological Information of DNA Types

**FIGURE 4:**
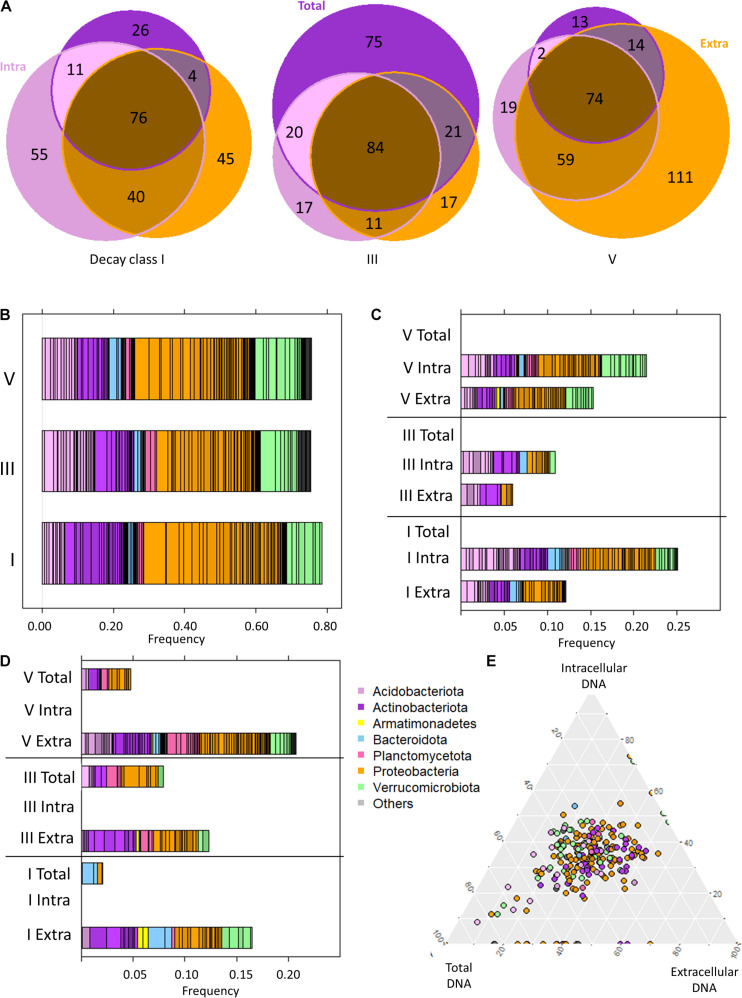
The bacteriome of *Picea abies* deadwood across decay classes (DCls I, III, V) and DNA types (extracellular and intracellular DNA fraction, total DNA pool). **(A)** Venn diagram illustrating the ASV occurrences among DNA types within each DCl. **(B)** Taxonomic composition of *P. abies* bacteriomes among DCls based on only the intracellular DNA fraction. **(C)** Taxonomic overview of ASVs that remained undetected in the total DNA pool within a DCl. **(D)** Taxonomic overview of ASVs that remained undetected in the intracellular DNA fraction within a DCl. **(E)** Ternary diagram of those ASVs with differential abundance among DCls (see Figure 2 and Supplementary Table 1). Their proportionate abundance among DNA types is illustrated.

On a broad(er) scale (than ASV or genera), the overall taxonomic distribution within a DCl was comparable between all DNA types (Supplementary Figure 2). The information about the bacteriome provided by the sequencing analysis of the intracellular DNA fraction, representing potentially living bacteria (intact cells), was similar to that depicted in the subchapter “*P. abies* deadwood bacteriomes differ among decay classes” (Figure 4B). At ASV level, there were distinct differences between DNA types for the different DCls (Figure 4A). We observed that *intra* and *in/ex* ASVs were taxonomically diverse (Figure 4C), and accounted for both a high number of ASVs (Figure 4A) and a significant percentage of reads (Figure 4C).

*Intra* and *in/ex* ASVs (Figure 3B) were frequently annotated as *Proteobacteria* in all DCls, except for DCl III. They mainly belonged to *Acetobacteraceae* (21 ASVs), *Beijerinckiaceae* (7 ASVs), and *Burkholderiaceae* (5 ASVs). Among *intra* ASVs, these taxa were frequent among all DCls (Supplementary Figure 3), underlining their relevance in the decomposition of *P. abies* deadwood.

The distribution of *intra* and *in/ex* ASVs annotated as *Acidobacteriota* followed a different pattern than that shown in the subchapter “*P. abies* deadwood bacteriomes differ among decay classes”. In DCl I, a higher number of these ASVs were affiliated to *Acidobacteriota* compared to *Actinobacteriota* and these ASVs were also higher in relative abundance (Figure 4C and Supplementary Figure 3). Several of them were consistently detected across DCls and also often across DNA types (Supplementary Figure 3). In DCls III and V, the *intra* and *in/ex* ASVs annotated as *Acidobacteriota* and *Actinobacteriota* had comparable numbers of ASVs and read proportions (Figure 4C). Underlining the high abundance and diversity of *Acidobacteriota* in DCl III, this taxon was higher in relative abundance in the total DNA pool compared to both the intra- and extracellular DNA fractions (Supplementary Table 2). The same observation was made for *Planctomycetota* (Supplementary Table 2), pointing toward an increased relevance of this taxonomic group in the intermediate stages of *P. abies* deadwood decay.

In DCls I and III, several ASVs annotated as *Verrucomicrobiota* were detected only in the intracellular DNA fraction (Figure 4C). In contrast to what has been reported in subchapter “*P. abies* deadwood bacteriomes differ among decay classes”, the majority of these ASVs were affiliated to *Chtoniobacteraceae* (6/8 ASVs). *Verrucomicrobiota* ASVs that remained undetected in the total DNA pool were usually present only in either the intra- or extracellular DNA fraction. Only in DCl V, *Verrucomicrobiota* ASVs undetected in the total DNA pool were found in both the intra- and extracellular DNA fractions (Figure 4B), and were mostly annotated as *Methylacidiphilaceae*.

Those ASVs not observed in the intracellular DNA fraction, but detected in the extracellular DNA fraction (*extra* ASVs) and in the total DNA pool (*tot/ex* ASVs) also (i) accounted for both a high number of ASVs (Figure 4A) and a significant percentage of reads (Figure 4D), and (ii) were taxonomically diverse (Figure 4D). Across all DCls, a relatively high fraction of *extra* and *tot/ex* ASVs belonged to *Actinobacteriota*. In DCl I, *Bacteroidota* and *Verrucomicrobiota* were frequently found in the *tot/ex* and *extra* ASVs. In DCl III and especially in DCl V, *Planctomycetota* appeared frequently detected with regard to *tot/ex* and *extra* ASVs.

In microbiome studies, statistical tools are often used to search for indicator ASVs that are interpreted as key species for a target ecosystem. Therefore, we evaluated if the indicator ASVs identified using Aldex2 were differentially detected among the DNA types. We plotted the read proportions of the ASVs across the three DNA types obtained from one log sample. We used the read proportion of the indicator ASVs in those groups of three samples and plotted their abundance for all logs. As the ternary diagram shows, the vast majority of indicator ASV proportions across logs was located in the middle of the triangle (Figure 4E), supporting that these indicator ASVs were mainly detected across all DCls and DNA types. In total, there was a number of 48 indicator ASVs. In DCl I, 26 out of 29 indicator ASVs were detected in all DNA types. In DCls III and V, the proportions were 23/29 and 28/34, respectively. Those points located on the lowest side of the triangle correspond to ASVs not being detected in the total DNA pool of a log. Moreover, within DCls, the relative abundances of these indicator ASVs differed between the DNA types (Table 2).

**TABLE 2:** Selected ASVs with significant differences between decay classes (DCl) calculated by Aldex2 generalized linear model.

	**Aldex2 model output**						**Raw reads**				**Median read proportion**									**Taxonomic annotation**	
**ASV**	**Estimate DC I**	**Estimate DC III**	**Estimate DC V**	**Pr(>| t|).BH DC I**	**Pr(>| t|).BH DC III**	**Pr(>| t|).BH DC V**	**median DC I**	**median DC III**	**median DC V**	**Venn (Figure 1C)**	**Intra_I**	**Extra_I**	**Total_I**	**Intra_III**	**Extra_III**	**Total_III**	**Intra_V**	**Extra_V**	**Total_V**	**phylum**	**genus**
19	0.03	7.83	7.86	1.000	0.015	0.015	0	91	108	III_V	n.d.	n.d.	0.62	0.97	1.32	0.64	1.13	1.29	1.50	*Actinobacteriota*	unknown IMCC26256
31	0.24	8.23	-2.00	1.000	0.029	0.998	0	206	0	III	0.35	n.d.	n.d.	2.43	1.39	1.88	n.d.	n.d.	n.d.	*Proteobacteria*	*Acidisoma*
43	-0.36	2.43	8.08	1.000	0.980	0.038	0	8	116	III_V	0.08	n.d.	n.d.	n.d.	0.21	0.13	1.10	1.22	1.20	*Actinobacteriota*	unknown
15	-1.39	1.80	9.75	1.000	1.000	0.012	0	0	184	V	n.d.	n.d.	n.d.	n.d.	0.68	n.d.	2.06	2.09	1.09	*Proteobacteria*	*Burkholderia*
34	8.39	-8.38	-10.35	0.008	0.044	0.015	121	0	0	I	1.79	2.79	1.91	n.d.	0.17	n.d.	n.d.	n.d.	n.d.	*Proteobacteria*	*Robbsia*
61	7.40	-7.54	-9.38	0.008	0.103	0.016	62	0	0	I	0.78	0.60	1.00	0.30	n.d.	n.d.	n.d.	n.d.	n.d.	*Acidobacteriota*	*Granulicella*
166	6.46	-4.85	-8.32	0.026	0.831	0.048	44	0	0	I	0.52	0.67	0.35	n.d.	0.09	0.14	n.d.	n.d.	n.d.	*Proteobacteria*	unknown *Acetobacterales*
230	5.95	-6.22	-7.78	0.028	0.247	0.055	32	0	0	I	0.24	0.48	0.26	n.d.	0.04	n.d.	n.d.	n.d.	n.d.	*Proteobacteria*	unknown *Caulobacterales*
16	-1.52	10.09	1.46	0.997	0.009	1.000	0	256	0	III	n.d.	n.d.	n.d.	1.48	1.06	2.60	n.d.	0.36	n.d.	*Acidobacteriota*	*Granulicella*
18	6.45	1.86	-6.82	0.031	1.000	0.110	68	166	0	I_III	0.92	1.21	1.12	1.74	1.71	1.72	n.d.	0.06	n.d.	*Proteobacteria*	*Endobacter*
27	7.89	-1.80	-4.94	0.001	1.000	0.708	86	82	14	core	1.00	1.00	1.15	0.99	0.77	0.65	n.d.	0.12	0.30	*Proteobacteria*	*Methylorosula*
42	6.21	0.87	-6.30	0.029	1.000	0.273	79	61	0	I_III	0.83	1.26	0.63	0.69	0.91	0.48	n.d.	0.24	n.d.	*Actinobacteriota*	unknown IMCC26256
48	-1.65	9.16	4.47	0.987	0.024	0.782	0	67	35	III_V	n.d.	n.d.	n.d.	0.77	0.84	0.55	n.d.	0.37	0.53	*Proteobacteria*	*Acidisphaera*
138	-1.80	8.67	2.56	0.977	0.013	0.961	0	65	0	III	n.d.	n.d.	n.d.	0.66	0.45	0.84	n.d.	0.04	0.08	*Cyanobacteria*	NA

*The output of Supplementary Table 1 was reduced to only ASVs that were not contained in the core ASVs of the different DCls (Figure 4A). For each of those ASVs, the Aldex2 estimate and Bonferroni-Holm corrected *p*-value of the Aldex2 model was reported. The median read number was indicated in order to show the size of the ASV. Their location in the overall Venn diagram was also shown. This can be interpreted as their respective center of mass. The median read proportion for each sample group was displayed to reflect in which group and to which extent these ASVs have been detected. n.d. = not detected.*

#### DNA Persists in the Extracellular Deadwood Environment

Given the fact that different DCls do not occur within the same log, at least not in the very same local point, and that the entire decay process is too slow to enable the sample collection of all DCls (I–V) from one deadwood log, the persistence of extracellular DNA in the logs and the exact abundances of ASVs’ intra- and extracellular DNA cannot be monitored over time. Therefore, we selected those ASVs that were detected in either DCls I and III or DCls III and V, respectively. Although we will likely miss a number of incidences for extracellular persistence using this approach, a signal in extracellular DNA persistence might be even stronger. Out of these 40 ASVs (24 + 16 = 40 ASVs, Figure 1C) that were detected in DCls I and III and DCls III and V, respectively, there were 19 ASVs whose relative abundance in the intracellular DNA fraction was lower in the more progressed DCl. Five of those ASVs were not detected in the intracellular DNA fraction of the more progressed DCl while they were found in the respective extracellular DNA and except for one ASV also in the total DNA pool. In a total of 16 ASVs their relative abundance was higher in the extracellular DNA fraction compared to the intracellular DNA fraction in the more progressed DCl. By overall rank abundance, that is ASV size measured by number of reads across all samples, these ASVs were listed at positions between 10 and 234, indicating that they were not ASVs of low-abundance. Moreover, several ASVs defined as indicator ASVs by ALDEx2 were detected in the intracellular DNA fraction of the less progressed DCl, then across DNA types and remained detectable in the extracellular DNA fraction in the more progressed DCl, i.e., after the ASV representing a specific population was no longer detectable in either the intracellular fraction or the total DNA pool (Table 2).

## Discussion

### Bacterial Richness

Assuming that more niches become available as deadwood decomposition progresses, we would expect that the number of ASVs detected increases with higher degree of decay that is from DCl I to V (Hunter, 1990). However, we did not observe an increase in ASV richness as a function of progressing deadwood decomposition. This was in line with the study on *P. abies* logs by Hoppe et al. (2015), who also applied sequencing methodology (454 pyrosequencing). Rinta-Kanto et al. (2016), in contrast, reported a higher bacterial richness in *P. abies* logs observed via microbiome sequencing data (454 pyrosequencing) and in line with this, a higher bacterial abundance was observed via quantitative PCR of 16S rRNA gene copy numbers as decomposition progressed. The increased richness with more progressed decay has also been confirmed by Kielak et al. (2016b), who researched decaying *Pinus sylvestris* logs. Tláskal et al. (2017), however, did not confirm an increased bacterial richness with progressing decay of different tree species, including *P. abies*. As the majority of α-diversity measures (richness), including the number of OTUs/ASVs is largely influenced by the number of samples, sequencing depth and data treatment, such as rarefying and filtering (Willis, 2019; Willis et al., 2019), an explanation for these controversial observations might be methodological rather than ecological. The sample sizes in the studies of Rinta-Kanto et al. (2016) and Tláskal et al. (2017) exceed the sample size of our study (*n* = 27), which is more comparable to the one from Hoppe et al. (2015). A higher number of samples per DCl, potentially even higher than that studied in Rinta-Kanto et al. (2016) and Tláskal et al. (2017) might further optimize the microbiome representability of the sample group and contribute to solving the riddle of decaying deadwood α-diversity. In addition to sample size, we hypothesize that richness estimations in microbial ecology suffer from an upper threshold of microbial units that can be observed in a sample. The reasons for this are a combination of the vast bacterial diversity known today, the patchiness of most ecosystems that are studied by pooled samples across physical space and unavoidable methodological limitations and restrictions, such as the amount of environmental sample used for DNA extraction, the template volume applied to PCR and the limited total number of amplicons subjected to sequencing. In addition, limited sample sizes, by means of low biological replications restrict the prediction of more reliable richness estimators. The increased depth of analysis obtained by the sequentially extracted DNA fractions of the total DNA pool clearly evidences the potential of the applied fine-tuning DNA approach, pinpointing that the statements on bacterial richness in natural ecosystems are problematic. Further investigations optimizing experimental design, sampling strategy, richness estimation, data analysis, and interpretation are necessary to make studies more comparable and a better representation of the ecosystem.

### The *P. abies* Deadwood Microbiome Is Diverse

Significant differences with regard to the bacterial community composition were expected among DCls according to their varying physico-chemical properties (Gómez-Brandón et al., 2017a). While pH, electrical conductivity and cellulose concentration decreased with increasing DCl, the moisture content as well as the concentrations of micro- and macronutrients such as N, Fe, and P increased (Gómez-Brandón et al., 2017a).

The vast differences in bacteriome compositions (Figure 1) reflected the vast ecological differences among deadwood at different decay stages. Despite the ability of some bacteria to degrade complex polymers, fungi are recognized as the main deadwood decomposers (Eastwood et al., 2011). The bacterial and fungal compositions are, therefore, likely interconnected within the deadwood habitat. As bacteria usually feed on the metabolites released by fungal activity, fungi may inhibit bacterial growth by the acidification of their environment and/or by the production of antibiotics (Folman et al., 2008; Johnston et al., 2016). With this in mind, acidotolerant, antibiotic resistant bacteria and those beneficial for the wood-inhabiting fungi are more likely to appear in higher quantities (Figures 1, 2). A high number of reads and a high number of ASVs were annotated as *Acetobacteraceae* (*Alphaproteobacteria*). Members of this taxon usually have an optimum pH ranging between 5 and 6.5 or even lower (Reis and dos Santos Teixeira, 2015), which might be advantageous for the fungi-dominated deadwood environment. Moreover, some members of this taxon are even able to fixate nitrogen (Reis and dos Santos Teixeira, 2015). Genes coding for enzymes involved in N-cycling, including N-fixation, have been found wide-spread across the bacterial phylogenetic tree (Nelson et al., 2016). Also some members of the taxa *Burkholderiales* and *Rhizobiales*, which were also found in high read proportions and numbers of ASVs (Figure 1), have been associated to this phylogenetically unconserved trait (Nelson et al., 2016; Shamseldin et al., 2017). In fact, members of these taxa have also been associated to methylotrophy (Chistoserdova et al., 2009), potentially giving them another selective advantage, especially with regards to co-inhabiting the deadwood together with fungi. Recently, Gómez-Brandón et al. (2020) showed that OTUs annotated as both *Rhizobiales* and *Burkholderiales* (*Gammaproteobacteria*) were associated to deadwood colonizing fungi in the very early stages of *P. abies* decay (2 years of decomposition). In their study, associations between mycorrhizal fungi and *Burkholderia* ASVs, which were also differentially abundant across DCls here (Figure 2), were reported. The changes in the fungal composition as a function of progressing decay (Arnstadt et al., 2016; Gómez-Brandón et al., 2020) are therefore likely to influence the bacteriome of the deadwood. In fact, this is reflected by the differences in relative abundance between DCls with regard to *Rhizobiales*, *Burkholderiales*, and *Acetobacteraceae* (Figure 2). The high selective pressure on bacteria in deadwood might result in a high turnover and a strong fluctuation of low-abundance populations, which is reflected by the high numbers and read proportions of ASVs that were detected in solely one DNA type (extracellular vs. intracellular fraction vs. total DNA pool) (Figure 4).

In addition to *Proteobacteria*, *Actinobacteriota* (peak abundance in DCl I), *Acidobacteriota* (peak abundance in DCl III), and *Verrucomicrobiota* (peak abundance in DCl V) were detected in our *P. abies* logs. These three phyla have been commonly found in bacterial communities colonizing deadwood from *P. abies* and other tree species (Sun et al., 2014; Hoppe et al., 2015; Kielak et al., 2016a; Rinta-Kanto et al., 2016; Tláskal et al., 2017; Johnston et al., 2019). Within *Actinobacteriota*, *Solirubrobacterales* accounted for the highest relative abundance in our study. Some members of *Solirubrobacterales* can act as lignin-degraders (Wilhelm et al., 2019), which might explain both their presence in all DCls and their peak abundance in DCl I.

In the case of *Acidobacteriota*, they peaked in relative abundance in DCl III. While *Acidobacteriota* ASVs were frequently detected in *intra* and *in/ex* (Figures 3B, 4C) and, therefore, likely reflect potentially living bacterial populations (intact cells), *Actinobacteriota* ASVs were frequently found in *extra* and *tot/ex* (Figure 4D) and are, therefore, potentially not viable. This latter phylum has been reported to occur on tree stems, including those from *P. abies* (Cregger et al., 2018; Ren et al., 2019). In our study, *Actinobacteriota* had a high ASV richness and to a certain extent read proportion in DCl I, suggesting that they might originate from populations that colonized the living tree, even though their metabolic role in *P. abies* deadwood degradation was probably minor owing to their pronounced presence in the extracellular DNA fraction. In fact, the capacity of some members of the *Acidobacteriota* to grow on cellobiose and N-acetyl-glucosamine and to produce enzymes required for chitin and cellobiose degradation has previously been shown for *Acidobacteriota* from forest soil (Lladó et al., 2016) and their adaptation to acidic conditions (Sait et al., 2006; Kielak et al., 2016a) might make some *Acidobacteriota* equally fit for DCl I and potentially more resistant to fungal competition than *Actinobacteriota*. The roles of these two phyla, their dynamics and their interactions with fungal deadwood degraders will help in understanding deadwood ecology and warrants deeper investigation.

In DCl V, a high number of ASVs and high read proportions were annotated to *Verrucomicrobiota*, exceeding both *Actinobacteriota* and *Acidobacteriota* (Figures 1C, 4B). Although *Verrucomicrobiota* are wide-spread across a range of environments, their vast majority remains uncultivated and is consequently, metabolically undescribed (Wagner and Horn, 2006). However, some few members isolated from high-temperatures, acidic environments have been shown to be methylotrophic (Wagner and Horn, 2006). As methylotrophy could provide an advantage in the deadwood environment due to fungal lignolytic activities that may result in the release of methanol, this trait might be spread across deadwood *Verrucomicrobiota*, thereby explaining their peak abundance in DCl V and both their high ASV richness and read proportions in the set of *intra* and *in/ex* ASVs (Figures 3B, 4C).

Despite their low proportions, *Planctomycetota*, mainly WD2101 soil group, were increasingly detected from DCl I to V (Figure 2). Several members of this taxon are known for degrading complex polymers, including cellulose (Wang et al., 2015), suggesting their potential role in deadwood degradation, which still remains underexplored (Tláskal et al., 2017; Probst et al., 2018). *Planctomycetota* have unique cell-wall and outer membrane structures as well as the ability to form biofilms and extracellular polysaccharides. Extracellular DNA has been shown to stabilize biofilms, to contribute to cell-cell-interaction within microbial populations and to serve as nutrient source (as reviewed by Pietramellara et al., 2009). In addition, extracellular DNA has been shown to increase *Planctomycetota* (WD2101) in soil (Morrissey et al., 2015). We hypothesize that the high concentration of extracellular DNA (Table 1) and the deadwood’s environmental approximation to soil with increasing decay might have benefited *Planctomycetota* abundance (Figure 4D) and might also contribute to the higher read proportions of *Planctomycetota* in DCl V compared to the other DCls and the higher numbers of unique *extra* ASVs observed in this DCl (Figure 4A).

It seems that multiple factors shape the microbiome of deadwood and determine its trajectory. Especially in terms of functional roles, more research involving the characterization, identification and description of isolates is necessary to shed light into the complex degradation process. In addition, co-cultivation experiments with wood inhabiting fungi should be performed in the future to study the interaction of deadwood inhabiting fungi and bacteria and to understand to what extent fungi shape the deadwood bacteriome.

### The Masking Effect of Extracellular DNA in *P. abies* Deadwood

The information about the bacteriomes derived from the three DNA types (extracellular vs. intracellular fraction vs. total DNA pool) followed the same trajectory (Figure 1). However, probably due to both methodological constraints and ecological aspects, they appear to differ in substantial details (Figure 4). The crucial impact of the rare biosphere on complex ecosystems is widely recognized (Jousset et al., 2017). The sequential extraction of the extra- and intracellular DNA fractions increased the ecological depth of analysis of the *P. abies* wood bacteriome, as compared to the total DNA pool (Figure 3). However, it often lowers the total amount of DNA extracted from the environmental material, due to repeated washing, ultimately resulting in the sum of intra- and extracellular DNA being lower compared to the total DNA pool (Table 1). Although repeated rounds of extraction from the very same sample can be applied in total DNA extraction procedures and may increase the microbial diversity observed by microbiome sequencing, the methodological differences between the total DNA pool and its extra- and intracellular DNA fractions (Wagner et al., 2015) suggest that analyzing these two fractions inherently results in greater depth (Figure 3). This observation was also reported for soil even with low-resolution molecular methods (Ascher et al., 2009; Chroňáková et al., 2013; Gómez-Brandón et al., 2017b), and for biogas slurries by using next-generation sequencing technologies (Nagler et al., 2018a; Nagler et al., 2020). From an ecological viewpoint, the extracellular DNA can be considered a natural product of microbial growth (Nagler et al., 2018a), including former ecosystem states. This latter aspect addresses the persistence of extracellular DNA in the environment as its degradation can be prevented by binding to various organic (e.g., cell debris) and inorganic, mineral colloids (soil components) or bacterial extracellular polymeric substances (Agnelli et al., 2007; Pietramellara et al., 2009; Nagler et al., 2018a). In agreement with this, we found a higher ASV richness in the extracellular DNA fraction compared to the intracellular one (Figure 1). Moreover, in line with the recent study by Nagler et al. (2020) on slurry bioreactors, we showed that DNA of certain bacterial populations remained detectable in the extracellular fraction, while it was not detectable in the respective intracellular DNA fraction, suggesting that these populations were no longer occurring in the monitored biological assemblage at the timepoint of sampling (Table 2). The conceptual differences between the extra- and intracellular DNA fractions imply that persisting DNA (extracellular DNA occurring in the extracellular environment) might partially mask intracellular DNA (methodologically extracted from intact cells via induced cell disruption) and thus obscure the snapshot of occurring microbes. Like the differential copy numbers of marker genes observed between different taxa, species-specific differences in DNA release might bias the composition observed in the extracellular DNA fraction and the total DNA pool. This potential masking effect of the extracellular DNA fraction over the intracellular one affects the assessment of the microbiome when directly analyzing the total DNA pool (made up by both DNA fractions), depending on its proportion with regard to the intracellular DNA fraction (Nagler et al., 2018a; Carini et al., 2020). For example, in soil and the marine environments up to 60% (Agnelli et al., 2004) and 90% (Torti et al., 2015) of the total DNA pool account for extracellular DNA, respectively. While the proportions of both DNA fractions are unknown for the majority of ecosystems, they were comparable in our *P. abies* deadwood samples (Table 1). They might even be patchy within ecosystems. Especially in terms of smaller operational units, single populations and rare biosphere, the proportions of DNA fractions and the potential masking effect of extracellular DNA should be considered. More studies on the masking effect of extracellular DNA in different ecosystems are necessary to confirm its generalizability (Nagler et al., 2020).

In our study, we found that the frequencies of several bacterial classes differed among DNA types (Supplementary Table 2). Our results suggest that the ecological role of *Acidobacteriota* in the decomposition of *P. abies* deadwood might be underestimated. Among the *intra* ASVs (Figure 3B), the diversity and abundance of *Acidobacteriota* was significantly higher than in the other two DNA types, especially in DCl I and also in DCl III (Figure 4). In addition, the abundance of indicator ASVs among DNA types fine-tuned their ecological interpretation (Table 2). In studies based on the total DNA pool, the composition of the extracellular DNA can mask the arrival, establishment and extinction of the ASV corresponding organisms despite “sufficient” sequencing/sampling depth, usually analyzed by rarefaction curves and sequencing coverage. In fact, the variations in bacterial abundances or even detection introduced by the potential masking effect of the extracellular DNA could complicate the assessment of microbiome-environment interactions. Consequently, the ecological role of organisms might be misinterpreted. Given the differential frequencies of bacterial classes in our study, this seems to hold true not only for species, but also for higher levels of taxa, which might change the perspective on a broader ecological scale. Due to the absence of indicator ASVs in the total DNA pool in some DCls (Table 2, Figure 4E), we might conclude that we do have statistical tools that can handle microbiome data, while we do not feed them with high-resolution microbiome data sets, which is not necessarily achieved solely by sequencing depth. Our findings provide further evidence that this can be accomplished with relatively little effort by the proposed fine-tuning DNA approach. Future studies can greatly benefit from studying different DNA types or at least considering their concept for a more accurate interpretation of molecular environmental data.

Despite the concerns raised regarding their conceptual differences, the DNA types studied here broadly resembled a comparable bacteriome and our results indicate that the differences in terms of composition with regard to their ecological interpretation might benefit scientific research. The analysis of the total DNA pool resulted in a robust, meaningful picture of the deadwood ecosystem. However, its resolution – independent of sequencing depth – is not as deep as the one provided by intracellular DNA, which is in agreement with Nagler et al. (2020) who made the same observation for slurry bioreactors. Especially if no exact time-depending compositional data are needed, the total DNA pool appears as a good choice, as it reflects the picture of the ecosystem including its former states. Studies focusing on microbial diversity or microbial populations with low-abundance might benefit from the increased sequencing depth provided by the intracellular DNA fraction. In these studies, the more complex extraction of intracellular DNA might be worth the effort. A general disadvantage of intracellular DNA analysis – equally to total DNA – by using commercial extraction kits, is the usually small amount of starting material. Studies on large and complex ecosystems – such as soil – can suffer from this, especially if they are heterogeneous and patchy. Analysis of the extracellular DNA can easily be up-scaled to larger amounts of input material and provide an efficient way of DNA extraction. However, ecosystems with high rates of microbial turnover and ecosystems underlying big disturbances or short-term successional changes might lack accuracy in terms of microbiome composition. Further studies are necessary to understand general concepts of DNA release into the extracellular environment. For evolutionary studies, it might be meaningful to analyze both DNA fractions of the environmental DNA pool. Extracellular DNA might be incorporated by living cells (natural transformation) thereby contributing to genome diversification and consequently, bacterial evolution/adaptation. Intracellular DNA can give a clear indication regarding which organisms diversify. In this context, metagenomic studies could prove especially valuable.

## Data Availability Statement

The datasets presented in this study can be found in online repositories. The names of the repository/repositories and accession number(s) can be found below: https://www.ncbi.nlm.nih.gov/bioproject/PRJNA682981/.

## Author Contributions

MG-B, JA-J, and MP organized the design of the study and performed the molecular wetlab work. MP analyzed the data. MP and MG-B wrote the manuscript. JA-J and HI edited the manuscript. MG-B and HI were PIs of this study. All authors contributed to the article and approved the submitted version.

## Conflict of Interest

The authors declare that the research was conducted in the absence of any commercial or financial relationships that could be construed as a potential conflict of interest.
