# Supplementary material for: The Molecular Information About Deadwood Bacteriomes Partly Depends on the Targeted Environmental DNA
Source: Front Microbiol. 2021 Apr 27;12:640386. doi: 10.3389/fmicb.2021.640386 (PMC8110828; doi:10.3389/fmicb.2021.640386)
Supplement: Supplementary file 3 [file Data_Sheet_1.docx]

**Supplementary Information**

**The molecular information about deadwood bacteriomes depends on the targeted environmental DNA**

Maraike Probst^1*^, Judith Ascher-Jenull^1^, Heribert Insam^1^, María Gómez-Brandón^1,2^

^1^Department of Microbiology, University of Innsbruck, Innsbruck, Austria

^2^Grupo de Ecoloxía Animal (GEA), Universidade de Vigo, Vigo, Spain


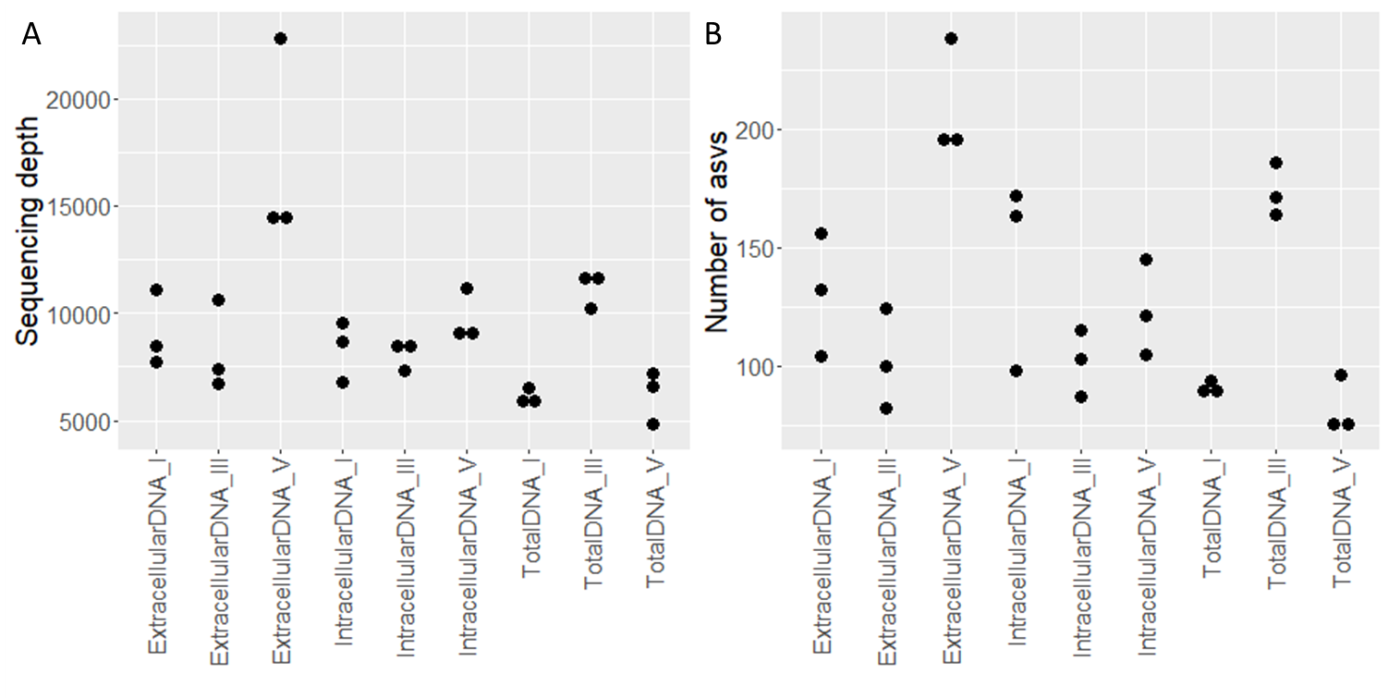


**SI Fig. 1 (A) Sequencing depth and (B) asv richness of the sample groups.**


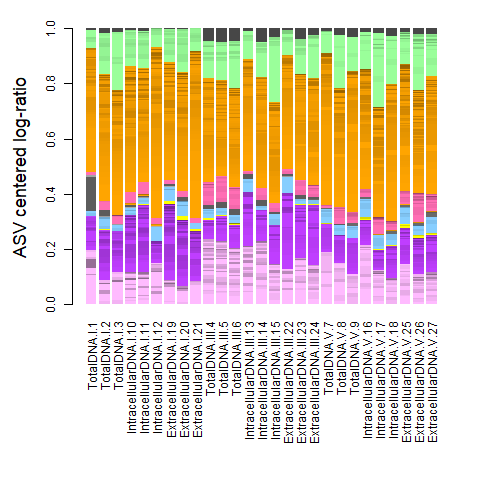


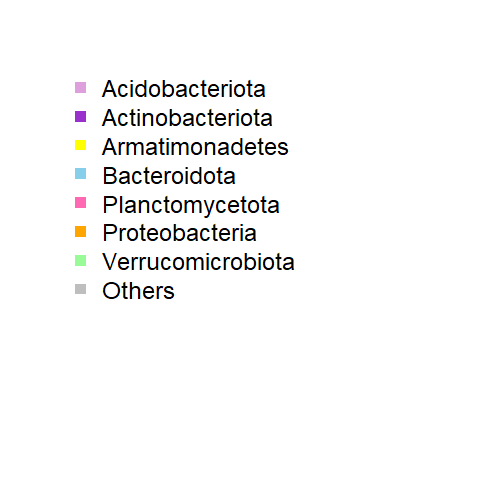


**SI Fig. 2 Overview of the deadwood bacterial dataset.** The V4 16S rDNA genes contained in the DNA extracted from the deadwood samples collected at different stages of decay (Hunter decay classes I, III, V) were sequenced on the Illumina Miseq platform. Raw data were filtered for quality (30 phred) using bbtools (http://jgi.doe.gov/data-and-tools/bbtools) and subsequently analysed using dada2 (Callahan et al. 2016). After error removal, the forward and reverse reads were merged. Merged reads were filtered for length (250-254 bp) and chimeric reads were removed. Taxonomy of asvs was assigned using silva reference v138 database and reads not belonging to bacteria, this is fungal and mitochondrial reads, were discarded. Using the barplot function of R package compositions (van den Boogaart et al. 2020), the centered log-ratio of each asv was illustrated; the option show.missing was set to false. Similar colour shades underline taxonomic annotation on phylum level.


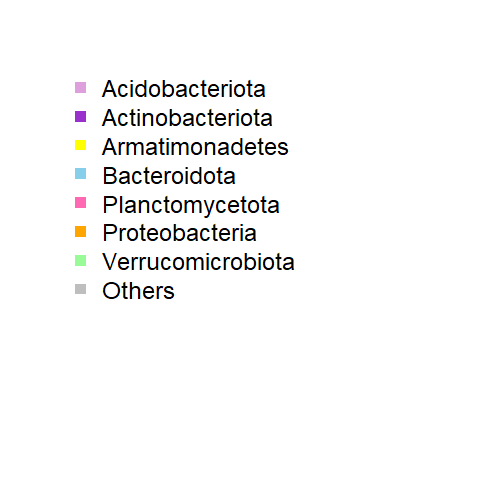

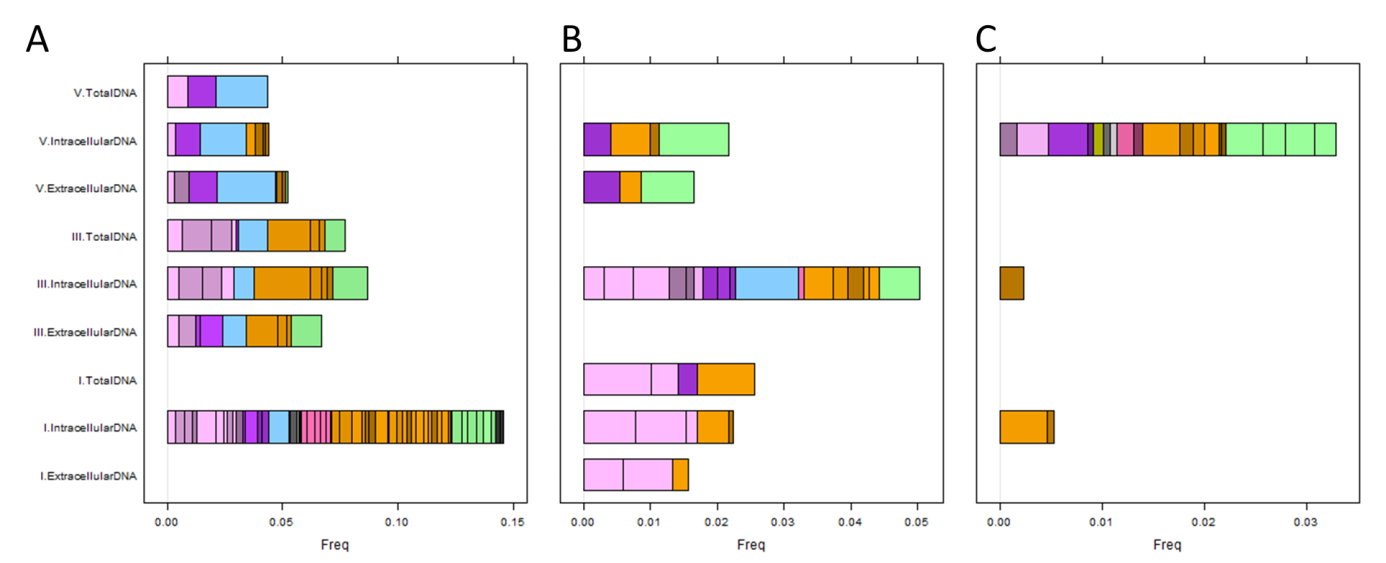


**SI Fig 3 Bacteriome composition of intracellular asv subsets from decay classes I (A), III (B) and V (C).** Following Fig. 4A, the relative abundances of those asvs were illustrated across all decay classes studied here that were detected exclusively in the intracellular DNA fraction at a respective decay class.

**Citations**

Callahan, B., P. McMurdie, M. Rosen, A. Han, A. Johnson & S. Holmes (2016) DADA2: High-resolution sample inference from Illumina amplicon data. *Nature Methods,* 13**,** 581-+.

van den Boogaart, KG, Tolosana-Delgado, R, Bren, M. 2020. compositions: Compositional Data Analysis. R package version 2.0-0. https://CRAN.R-project.org/package=compositions
